# An Emulsion System Based on a Chip Polymerase Chain Reaction

**DOI:** 10.3390/molecules13123057

**Published:** 2008-12-09

**Authors:** Qinyu Ge, Pinfei Yu, Yunfei Bai, Zuhong Lu

**Affiliations:** 1 Key Laboratory of Child Development and Learning Science, Ministry of Education, Southeast University, Nanjing, 210096, P.R. China; E-mails: geqinyu@seu.edu.cn (Q-Y. G.), pinfyu@gmail.com (P-F. Y.); 2 State Key Laboratory of Bioelectronics, Southeast University, Nanjing, 210096, P.R. China; E-mail: whitecf@seu.edu.cn (Y-F. B.)

**Keywords:** PCR amplification, Emulsion, Immobilized primers

## Abstract

In this paper we describe a novel method for detecting many DNA fragments through efficient amplification by using an emulsion system based on “on-chip” PCR instead of conventional multiplex polymerase chain reaction (PCR). During the preparation of on-chip PCR, a set of primers were immobilized on a slide and other sets were in an emulsion system. Different emulsion phase primers and other related PCR components were dispersed in different droplets of the emulsion system, and then, due to the thermal instability of emulsion droplets, they would be released onto the surface of the slide after preheating in the first PCR step. To test the above method, we used plasma DNAs from pregnant women who was carrying a male fetus for gender identification. Four different Y chromosome DNA fragments were selected. Results showed that different DNA fragments could be simultaneously amplified with satisfactory results. It is suggested that a simple, convenient and inexpensive on-chip PCR method has been developed.

## Introduction

Multiplex PCR, along with PCR, is one of the most commonly used techniques in molecular biology. It is an essential cost-saving technique for large scale genotyping with significant scientific, clinical, and commercial applications, including gene expression [1], whole-genome sequencing [2], facilitating the diagnosis of infectious diseases [3] and forensic analysis, including human identification and paternity testing [4]. It often serves as the first step in many genetic analysis methods and a number of analytical methods are available for the detection of PCR products. These methods, and at the same time one of the most labor-intensive, require the polyacrylamide or agarose gel electrophoresis of the PCR products, followed by blotting onto a membrane and hybridization to detectable labeled PCR product-specific probes. However, the most specific problem of multiplex PCR is the interference between primers and other components in the amplification system, which is always a problem that is not completely solved although many approaches have been proposed, such as solid phase PCR [5, 6]. The conventional solid phase PCR often requires extra equipment such as frames for sealing the solution, 96-well polystyrene plates as a solid support, or beads in solutions for oligonucleotide immobilization [7, 8]. The method described in this paper employs and emulsion system and conventional slides with simple modification. We have attempted to develop an emulsion based on-chip polymerase chain (EC-PCR) reaction in this study.

Emulsion PCR is one of the recently developed techniques with higher sensitivity and amplification efficiency which is widely used in much research [9,10,11,12,13]. Most of those typical Next Generation Sequencing Systems, such as 454, SOLiD, etc., take advantage of this technique. Despite its superiority, however, thermal instability of emulsion PCR is still an intractable problem which afflicts bench researchers. Many PCR failures can be attributed to the thermal instability of the emulsion system, for which a crucial factor is the kind and ratio of stabilizer. In this study, however, thermal instability of emulsion system was utilized as a positive factor in the newly proposed method of on-chip PCR. Different free primers and other PCR components are dispersed in different droplets of emulsions before the thermal cycle, and then they will be released to the surface of slides with previously immobilized primers, which is followed by PCR amplification on the slide. These procedures are supposed to eliminate the problem of primers interference that is common to conventional multiplex PCR. 

The discovery of fetal DNA in maternal plasma of pregnant women is one of important progresses in human genetics [14,15,16]. In present study, for method validation four different fragments distributed on the Y chromosome were detected from plasma of pregnant women who were carrying a male fetus. 

## Results

### Strategy of EC-PCR

**Figure 1:**
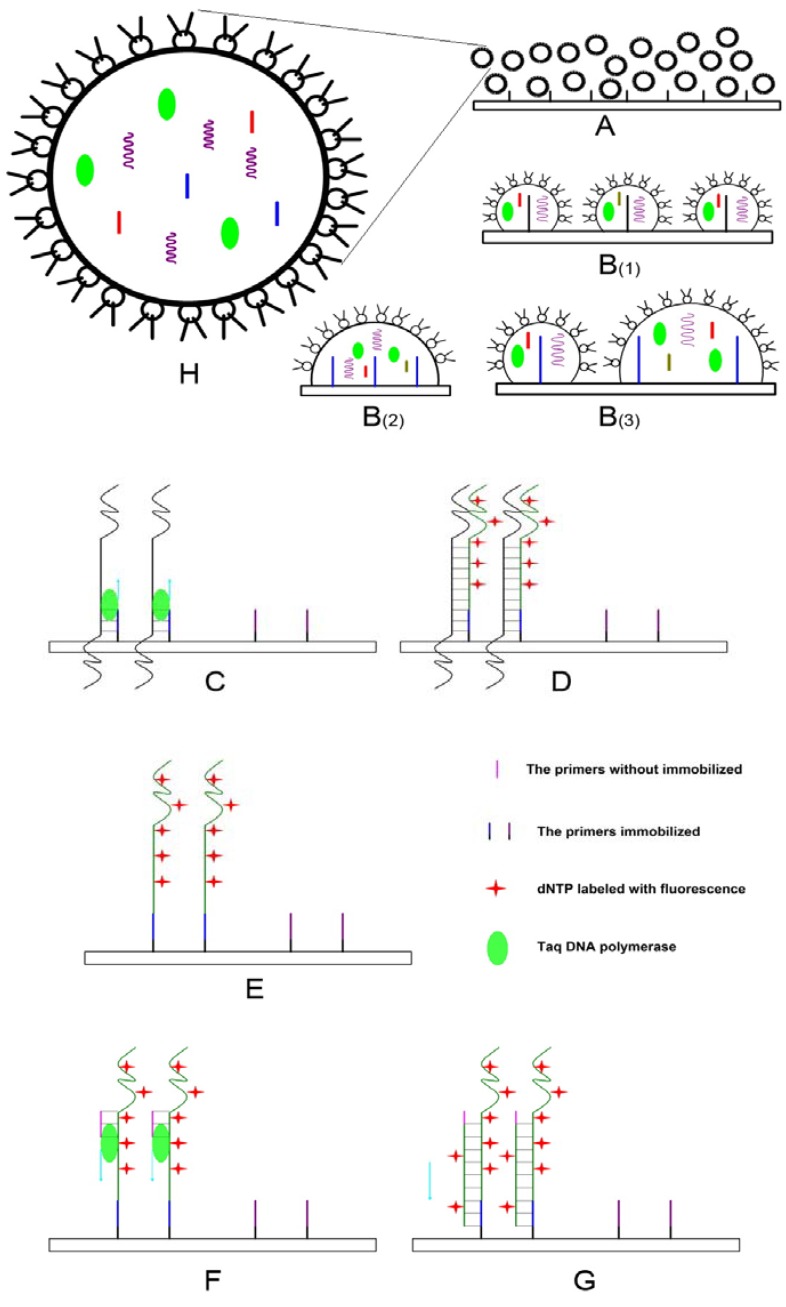
The EC-PCR strategy.

Figure 1 is a schematic diagram illustrating EC-PCR. Before the thermal cycle, an emulsion system containing one of the PCR primer pairs and other components was prepared; the slide was cleaned and modified for the immobilization of the other primer. The on-chip PCR can be carried out after simply adding 20 μL of emulsion to the slide surface. After initial denaturation for 10 min at 95°, the free primers and other components are released gradually and the thermal cycles started. During the first cycle, denatured target DNA anneals to primers that are covalently bound on their 5’ ends to slides. Primers are extended on their free 3’OH ends during the extension phase. 

In the picture A denotes the first step of on-chip PCR, when emulsion was simply added to the surface of slide. B(1), B(2) and B(3) show three possible emulsion phase primer states and the other PCR component have been released to the slide. C to G described the role of primers and template on the slide in the first two cycles of on-chip PCR, Cy5 labeled dUTP was incorporated at each extension step. H denotes the typical water in oil emulsion system.

During the second PCR cycle, extension products from covalently bound primer are hybridized to complementary primers which are free on the surface of slides. After the second extension cycle, double-stranded PCR products are covalently bound to the slides at both of their 5’ ends. During the third and all subsequent denaturation, annealing, and extension cycles, additional PCR products are generated on the solid phase until primers are consumed. The localized primers and the emulsion phase primers provide each template with a minimal-complexity and decrease the likelihood of nonspecific priming and product cross-reactivity that normally occurs during multiplexed amplification. Cy5 labeled dUTP was incorporated at each extension step which could be detected by laser scanner.

### Thermal instability of certain emulsion system

**Figure 2:**
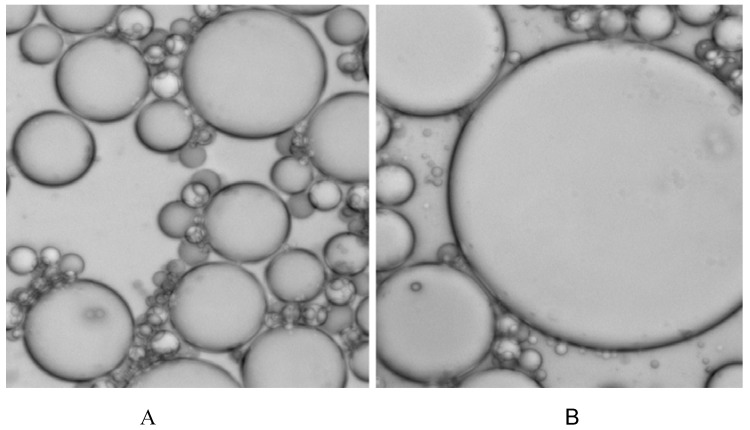
Thermal instability of certain emulsion system.

The emulsion system was prepared according to the previous study by Williams *et al*. with some modification [9]. With this specific ratio of stabilizer in oil phase of emulsions, phase transition occurred and many of the droplets in the system integrated and broken gradually when heated to 95°C for about 10 min. Figure 2 shows the emulsion photo captured by a Nikon E200 microscope under a 40×lens before (A) and after (B) heating. In the figure we can see that many droplets broke and integrated and their diameter increased noticeably. 

### EC-PCR from maternal plasma DNA

**Figure 3:**
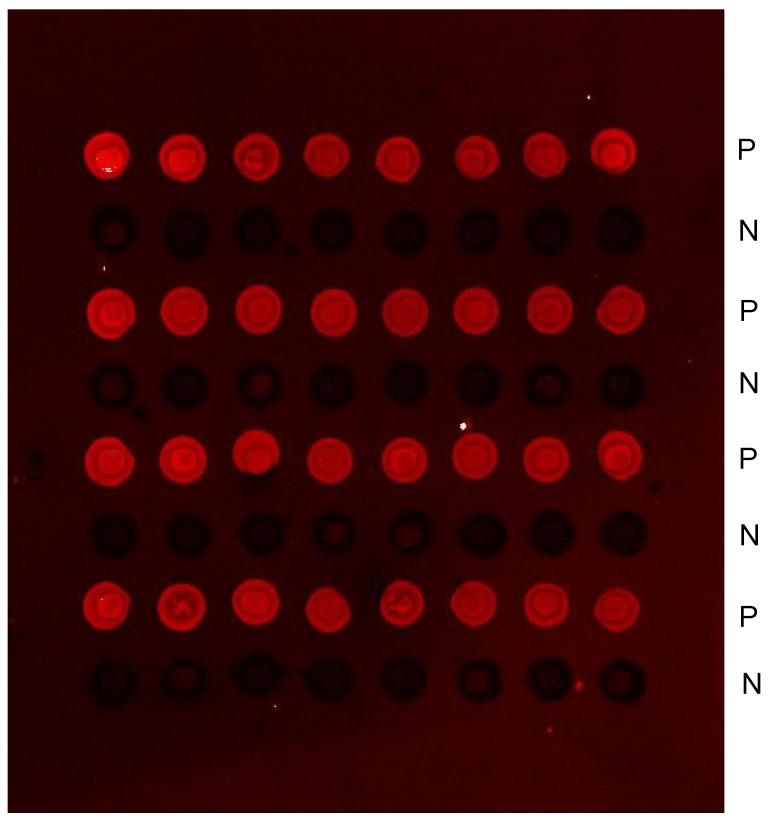
Reliability of the EC-PCR.

We next attempted to determine whether emulsion based on-chip amplification using locus-specific primers was reliable and sensitive enough to amplify conventional DNA templates such as maternal plasma DNA. We selected SRY gene as an example for study. The reverse primer was spotted on the slide and immobilized, and another unrelated primer was also spotted alternately on the slide as a negative control. Eight repeats were spotted in each row. The amplified results could be seen in Figure 3, no signal was obtained in the negative control spots. As PCR is an exponential process, that can be described by the equation N = N0 (1 + E)n, where E defines the efficiency of amplification, N0 is the initial number of target molecules, and N is the amount of product synthesized [17]. Amplification reactions at early and intermediate cycle numbers were used to determine that the efficiency of EC-PCR was near 0.6 (data not shown), whereas typical solution-phase amplification generally has an efficiency of 0.8. Additionally, the effect of primer concentration on product yield also has been examined. No product is detected when the primer concentration is less than 10 uM. Product is detectable at a primer concentration of 20 uM, but increases with 80 uM primer concentration, all these results in the study is obtained at primer concentration of 80 uM. 

The spots in the picture show the amplification result of the positive and negative primers on the slide. P denoted the spots of each row were positive primer and N denoted the spots in the row were negative primer.

### Multiplexed EC-PCR

**Figure 4:**
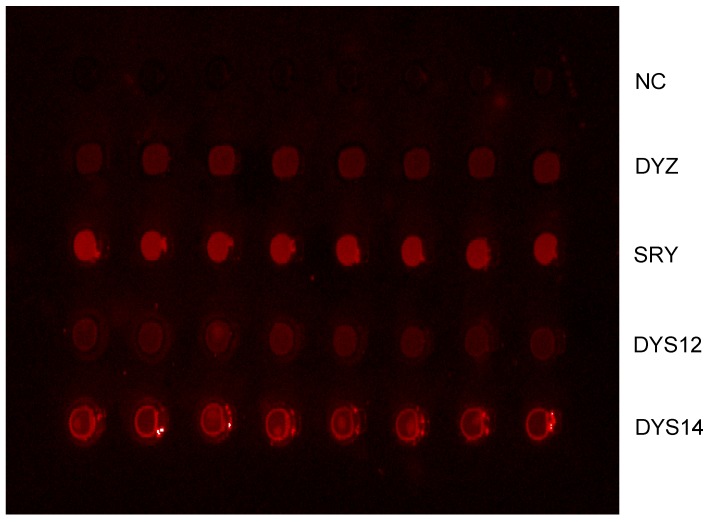
Results of fetal sex identification by the EC-PCR.

To evaluate the ability of EC-PCR to reduce the interference between primers when multiplexed, we performed an EC-PCR reaction to detect four different Y chromosome genes. The primer sequences in this study were not chosen or optimized for their ability to enable solid-phase or solution-phase amplification. The DNA sequences of the primers used in this study showed in Table 1. All four sites were amplified and positive signals were obtained from the primers array on the chip, and no signals were seen in the negative control spots. Same result was obtained from three repeats of each sample, which confirmed the reproducibility of EC-PCR. The fetal sex were identified correctly with this method in all except one of the samples collected from pregnant women whom carrying a male fetus at 35 days of gestation. Among the 22 samples, two of them were not fully detected in the four genes studied (Table 2), DYS12 was not obtained positive signal in one sample and the other is DYZ. “×” in the table showed the site obtained negative result in EC-PCR amplification. The actual gender of the fetus which their mother’s plasma samples were studied here was confirmed when delivery. Fig.4 showed the typical result of fetal sex identification from maternal plasma with EC-PCR. 

**Table 1:** DNA sequences of the primers used in this study.

Name		DNA Sequences (5’ to 3’)	5’Modification	GenBank No.
SRY	462F	(T)_6_GCAGGGTACCGAAGAGGGA	amido	gi:785026
	210R	(T)_6_GTCTCGCGATCAGAGGCGCAAGA		
DYS12	423F	(T)_6_ACTTCCCTCTGACATTACCTGATAATTG	amido	gi:245973
	172R	GTCATAGAAGAGTCAAGTCAGTCA		
DYS14	637F	(T)_6_GCCAGGAAGGCCTTTTCTCGGCA	amido	gi:38003
	880R	TTCCCCTTTGTTCCCCAAA		
DYZ	33642F	(T)_6_GTGGATTCATCTCACAGAGTTAAA	amido	gi:29824712
	33259R	ACACATCACAAAGAACTATG		

**Table 2:** Fetal sex identification results of the 23 samples detected by EC-PCR.

Samples	Gestation Ages	Signals obtained in the sites detected			
		SRY	DYS12	DYS14	DYZ
1	54 Days	√	√	√	√
2	53 Days	√	√	√	√
3	42 Days	√	√	√	√
4	61 Days	√	√	√	√
5	44 Days	√	√	√	√
6	44 Days	√	√	√	√
7	57 Days	√	√	√	√
8	55 Days	√	√	√	√
9	45 Days	√	√	√	√
10	56 Days	√	√	√	√
11*	52 Days	√	×	√	√
12	55 Days	√	√	√	√
13	61 Days	√	√	√	√
14	43 Days	√	√	√	√
15	48 Days	√	√	√	√
16**	35 Days	×	×	×	×
17	47 Days	√	√	√	√
18	65 Days	√	√	√	√
19	74 Days	√	√	√	√
20*	40 Days	√	√	√	×
21	60 Days	√	√	√	√
22	51 Days	√	√	√	√
23	46 Days	√	√	√	√

* sample which the four sites not all detected ** sample which obtained false negative result

NC = signal obtained from negative control primer; the four rows of spots below the control in the picture denoted the amplification result of DYZ, SRY, DYS12 and DYS14 respectively.

### Non-emulsion control

**Figure 5:**
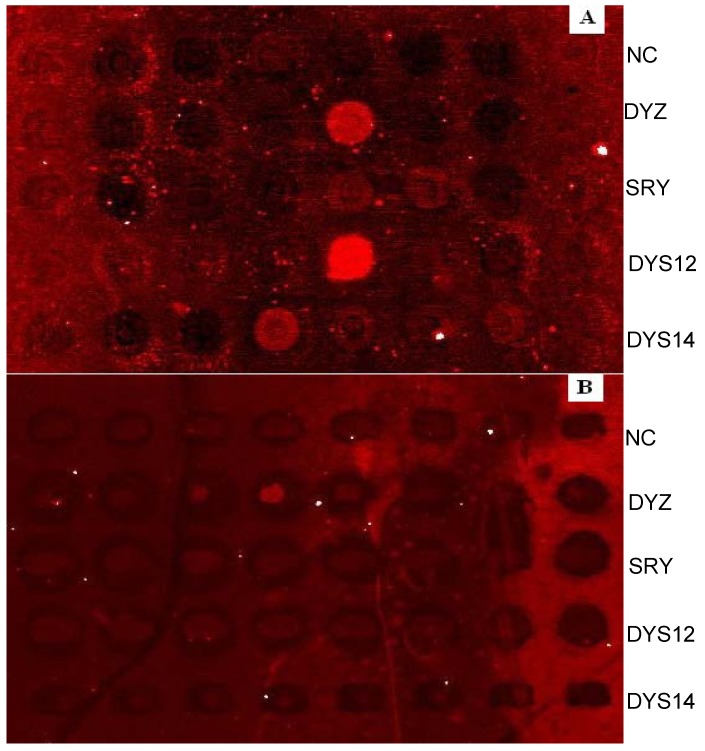
Comparing results of non-emulsion based on chip PCR.

To evaluate whether the emulsion system played a key role in our solid phase amplification, a control experiment in which only mineral oil was added in PCR system was carried out, because one of the main advantages of EC-PCR is the emulsion system could prevent the evaporation of amplification component during thermal cycles. The results can be seen in Figure 5 below. The arrangement of the primer arrays was same as Figure4, where there are five rows of spots and those were negative control, DYZ, SRY, DYS12 and DYS14, respectively, from top to bottom. We also compared the amplifications with and without the cover glass. A in the figure shows the result of amplification system covered with a cover glass, many noise signals and higher background signals were found in the chip comparing with the emulsion based solid phase amplification. Furthermore, positive signals were inconsistent and found only in few of the primer spots; similar higher background signals were obtained in B of Figure 5 which no cover glass was used during PCR process, and very low density and equivocal signals were found in the chip. 

## Discussion

In this study, an EC-PCR amplification method was proposed. It provides a high throughput assay platform for biological and medical researches that takes advantage of the ability to simultaneously amplify multiple loci in a single reaction. The multiplexed amplification was carried out on a slide, thereby greatly reducing the local complexity of primers and eliminating artificial priming events and primer-primer in the reactions. Furthermore, the decrease of interferences also contributed to the solution phase primers in the droplets of emulsion system where all molecules required in PCR is separated before thermal cycle. This method overcomes the critical bottlenecks faced in conventional high throughput analysis; that is the ability to perform multiplexed amplifications without having to add a complex of primers.

We have combined the sensitivity of PCR with the capability to multiplex different immobilized primers in a single reaction. Because the primers are immobilized to the slide surface through the 5’ end during amplification, PCR product is synthesized on the slide itself, thereby reducing the total primer complexity that would occur if primers were present free in solution. This minimizes the interactions between primers from different loci during multiplexed reactions, which can contribute to unwanted products such as primer-dimers or nonspecific amplification products. The efficiency of on-chip PCR (0.6) was relatively low compared to conventional PCR, which might be ascribed the fact that one of the primers are held in the surface of the slide, thus decreasing the chance of hybridized with template DNA in each cycle of amplification. The results in Figure 5 show that no satisfactory results were obtained when no emulsion was used in the solid amplification system and this indicates that emulsions did play important role in our on-chip PCR amplification.

The sex identification from maternal plasma results indicated that the sensitivity of the EC-PCR was acceptable, as it has the ability to detect fetal DNA in early stage of gestation as well as conventional PCR. The only one sample with false negative detection result was collected at 35 days of gestation; the other two samples which not fully detected in the four sites were also collected in very early stage of gestation (one is 40 days and the other is 52 days). Conventional PCR and real time PCR was also have very low sensitivity at this gestation stage [18]. This may be ascribed to the fact that very little fetal DNA is present in maternal plasma at an early gestation age stage.

Operation of this EC-PCR amplification is very simple. Only one step of the preparation of emulsions and addition to the slide is required before placing it on a thermal cycler; no gel electrophoresis and radioactive material is required for detection of PCR products; the signals of amplification results on the slide can be scanned under a laser scanner after simply washing. In the whole process, no expensive material or device is consumed, the components for emulsion preparation are very cheap and a conventional cleaned slide is enough for modification in this method. We anticipate that with this emulsion based system, many types of research could be carried out, such as simultaneous genotyping of many SNP, detection of the methylation status of whole CpG island and almost all the on chip reactions that need thermal cycles. 

## Conclusions

In conclusion, we have developed a high-throughput detection system that takes advantage of the ability to perform multiplexed amplified reactions on a slide with an emulsion system. It has been successfully applied to the detection of fetal sex from plasma of pregnant women. It is suggested a simple, convenient and inexpensive method which could be used in high throughput genotyping, methylation detection and other related research.

## Experimental

### General

The peripheral blood samples used in this study were collected from Nanjing Zhongda Hospital. All the primers and probes were synthesized and purified by Invitrogen Inc. (Shanghai, P.R. China). The PCR system and Cy5 labeled dUTP were purchased from TaKaRa Bio Company (Shiga, Japan). Deionized distilled water was purified by Milli-Q® (Millipore Corp., Billerica, MA, USA). 20 × saline-sodium citrate (SSC) buffer was prepared as follow: NaCl (35.06 g) and sodium citrate (17.64 g) were dissolved in deionized distilled water (180 mL); the pH was adjusted to 7.0 with NaOH, the solution was made up to 200 mL and then autoclaved before use. To use 20×SSC, it was simply diluted with an appropriate amount of distilled/deionized water. The EC-PCR was carried out under a tower thermal cycler (PTC220, Bio-Rad). All the slides used in this research were scanned by using a LuxScan10K Scanner (Capitalbio, Beijing, P.R. China) in the Cy5 channel at 85% laser power, 80% PMT gain, 5 μm resolution. 

### Sample preparation

Twenty three peripheral blood samples were collected from pregnant women at first trimester for test in this study. Plasma DNA was extracted from plasma samples (200 μL) using the QIAamp Blood Mini Kit (Qiagen, Hilden, Germany) following the blood and body fluid protocol supplied by the manufacturer. Plasma DNA was eluted into a final volume of 20 μL and 2 μL samples was used as templates for PCR analysis. 

### Preparation of slides and EC-PCR

The slide was cleaned and modified with aldehyde according to the method described in our previous study [19]. Emulsions for on-chip PCR were prepared by some modifications of described methods [9, 20]. The oil phase was composed of 7.5% Span 80 (no. S6760, Sigma) and 1.0% Tween 80 (no. S-8074, Sigma) in mineral oil (no. M-3516, Sigma). The components of aqueous phase were consistent with conventional PCR except higher concentration of MgCl_2_ (final concentration was 25mM). On-chip PCR was carried out under the following cycling conditions: 95°C for 10 min, 40 cycles of 94°C for 30 sec, 56°C (or corresponding with annealing temperature) for 30 sec, and 70°C for 30 sec. During extension step, Cy5 labeled dUTP instead of dTTP incorporated in each newly synthesized strand. Thermal stability of this emulsion system was detected by heating to 95°C for 10 min; the photo was captured under a microscope (Nikon E200) before and after heating for comparison.

### Multiplex detection and scanning

Four genes (SRY, DYS12, DYS14, and DYZ) distributed on Y chromosome was selected for test in this study. All the reverse primers were modified with NH_2_ on their 5’ ends (DNA sequences not showed). After PCR thermal cycle, the slide was cleaned with 2× SSC (1× SSC is 0.15 mol/L NaCl plus 0.015 mol/L sodium citrate), 0.1% sodium dodecyl sulfate (SDS) at 50°C for 5 min, 0.2× SSC,0.1% SDS, and distilled water at room temperature for 2 min in sequence and were then was dried for scanning. Non-emulsion control experiment was also carried out for comparison in the study.
